# Three-Dimensional Imaging of Hepatic Sinusoids in Mice Using Synchrotron Radiation Micro-Computed Tomography

**DOI:** 10.1371/journal.pone.0068600

**Published:** 2013-07-05

**Authors:** Yae Jin Yoon, Soeun Chang, Oh Youn Kim, Bo-Kyeong Kang, Jaesung Park, Jae-Hong Lim, Jung Yun Huang, Yoon-Keun Kim, Jae Ho Byun, Yong Song Gho

**Affiliations:** 1 School of Interdisciplinary Bioscience and Bioengineering, Pohang University of Science and Technology, Pohang, Republic of Korea; 2 Department of Materials Science and Engineering, Pohang University of Science and Technology, Pohang, Republic of Korea; 3 Department of Life Sciences, Pohang University of Science and Technology, Pohang, Republic of Korea; 4 Department of Radiology and Research Institute of Radiology, University of Ulsan College of Medicine, Asan Medical Center, Seoul, Republic of Korea; 5 Department of Mechanical Engineering, Pohang University of Science and Technology, Pohang, Republic of Korea; 6 Pohang Accelerator Laboratory, Pohang University of Science and Technology, Pohang, Republic of Korea; Mayo Clinic College of Medicine, United States of America

## Abstract

Hepatic sinusoid, the smallest vessel in the liver, plays important roles in hepatic microcirculation. Although the structure of the hepatic sinusoids affects diverse functions of the liver, little is known about morphological alterations in the sinusoids under pathological conditions. In this study, we show that the structure of hepatic sinusoids can be identified three-dimensionally in normal and carbon tetrachloride-injured mouse liver, using the absorption mode of synchrotron radiation micro-computed tomography. We observed that the hepatic sinusoidal structure on tomographic slice images was similar to that on histological images of normal and acutely injured mice. Moreover, centrilobular necrosis and structural alterations of the sinusoids in the necrotic region were detectable on tomographic slice and volume-rendered images of the acutely injured mice. Furthermore, quantitative analyses on 3D volume-rendered images of the injured sinusoid revealed decrease in the volume of the sinusoid and connectivity of the sinusoidal network. Our results suggest that the use of synchrotron radiation micro-computed tomography may improve our understanding of the pathogenesis of hepatic diseases by detecting the hepatic sinusoids and their alterations in three-dimensional structures of the damaged liver.

## Introduction

Hepatic sinusoids are capillary bed in the liver consisted with sinusoidal endothelial cells, stellate cells, and Kupffer cells [1]. Nutrient-rich venous blood and oxygen-rich arterial blood combines and flows through the sinusoids [2]. Sinusoids are separated from adjacent hepatocytes by the space of Disse [3]. The sinusoids run straight between the liver cell cords and communicate with each other though interconnecting sinusoids [3,4]. The diameter of hepatic sinusoids is 7-15 µm and the diameter of pericentral sinusoid is larger than that of periportal sinusoid [3–5]. The hepatic sinusoids are special in that they possess the sieve plate-like pores [3,4,6]. Unlike non-hepatic capillaries that exchange molecules ranging between 0.5 and 12 nm by endocytosis and transcytosis, hepatic sinusoidal endothelium could exchange molecules ranging up to 150-175 nm through the sieve plate-like pores [6,7].

Under normal physiological conditions, the ingested toxins are transported into hepatocytes and detoxified by penetrating the sinusoids of the hepatic microcirculatory system [3]. However, under pathological conditions, morphological changes in the sinusoids impair hepatic microcirculation and induce functional damage [8–12]. Recently, structural alterations such as the diameter, surface area, and fenestrae of the sinusoids have been reported in acute and chronic liver injury and liver cancer [8,9,12]. However, there still lack quantitative nondestructive imaging studies on normal and diseased liver models to reveal the structural difference.

With increased interest in the significance of the hepatic sinusoids, significant efforts have been made to investigate sinusoidal structure, but light and confocal microscopic studies on the sinusoids are incomplete compared with nondestructive three-dimensional (3D) imaging [11,13,14]. To date, nondestructive structural studies of hepatic microvasculature have been performed by micro-computed tomography (micro-CT) using a conventional X-ray source. Conventional micro-CT nondestructively acquires 3D images from thick tissue; however, the image resolution is insufficient for observing the sinusoids. These studies, therefore, are restricted to the hepatic biliary, arterial, and venous systems [15–17].

Synchrotron radiation micro-CT provides spatial resolution of 1 µm and is a feasible imaging system for studying sinusoids and their alterations in 3D space [18–20]. Recently, 3D images of the cortex microvasculature in the brain at 1 µm resolution have been visualized, and the differences between wild-type and APP23 knockout mice were successfully observed [20]. Considering the diameter of the capillaries in the mouse brain, the spatial resolution of synchrotron radiation micro-CT is sufficient to detect the sinusoids with diameters of 7-15 µm in the mouse liver [3,4]. The present study applied synchrotron radiation micro-CT to investigate the 3D structure of hepatic sinusoids and their alterations associated with acute liver injury.

## Materials and Methods

### Materials and animals

Carbon tetrachloride (CCl_4_), corn oil, propylene oxide, and phosphate buffered saline (PBS) were purchased from Sigma (St. Louis, MO). Osmium tetroxide (OsO_4_), glutaraldehyde, and epoxy resin were obtained from Electron Microscopy Sciences (Fort Washington, PA).

Male, 6-week-old C57BL/6 mice were maintained under specific pathogen-free conditions. All animals received humane care, and the experiments were approved by the Institutional Animal Care and Use Committee at Pohang University of Science and Technology, Pohang, Republic of Korea (approval number: 2011-01-0015).

### CCl_4_-induced liver injury and specimen preparation

The CCl_4_ was prepared as a 50% (vol/vol) solution in corn oil, and a single dose of 2 ml/kg of body weight was administered by intraperitoneal injection (n = 5) to induce hepatic injury [21,22]. Control animals were injected with corn oil alone (n = 5). Groups of age- and sex-matched mice were sacrificed on day 1 after acute CCl_4_ treatment. Liver tissues were obtained from five mice in each group at the indicated times and then fixed with 2.5% glutaraldehyde in PBS. The right hepatic lobe from each mouse was processed for histological analysis and synchrotron radiation micro-CT. Liver sections (5 µm) were stained with hematoxylin and eosin for general histology and examined under a light microscope. For synchrotron radiation micro-CT, liver sections (2-3 mm) were stained in 1% OsO_4_ for 4 h, dehydrated in an ethanol series, and embedded in an epoxy resin in 1.5 ml Eppendorf tubes.

### Instrumentation

All experiments were performed with polychromatic X-ray beams (4-15 keV energy range) at the 7B2 X-ray microscopy beamline of the Pohang Light Source (2.5 GeV), Republic of Korea [23]. A silicon shutter was used as an attenuator to control total X-ray flux. The detector system consisted of a thin (100-250 µm) and cleaved CdWO_4_ single crystal scintillator and a charge-coupled device (CCD)-based video camera. The image displayed on the scintillator was converted to a visible image and then magnified by an optical lens before it was captured by the CCD. Specimens embedded in resin were mounted on a sample holder and typically placed 100-200 mm apart from the detector to achieve optimum contrast. Scanning was performed by rotating the specimen in increments of 0.18^o^ in an X-ray beam and acquiring an X-ray transmission image at each angle of view. Three-dimensional image reconstruction from 1000 projections was carried out using a standard filtered back projection algorithm (Image Pro Plus; Media Cybernetics, Silver Spring, MD). Reconstructed tomographic slice images consist of 1600 × 1200 pixels in the horizontal and vertical directions and the horizontal field of view is 580 µm. Using synchrotron radiation micro-CT, images with voxel size of approximately 1 µm are achievable [23,24].

### Analysis of the reconstructed image data

As the X-ray images in this study contained both hepatic microvasculature and surrounding parenchymal cells, the desired area to be displayed and analyzed was segmented. Using the segmentation application of Amira software (Mercury Computer Systems, Chelmsford, MA), each pixel of the image was assigned a label describing the region to which it belonged. Pixels in tomographic slices were denoted as parenchymal area, sinusoidal area, central venous area, or portal venous area. Each labeled region was surface generated for 3D visualization using the Amira software based on human-computer interaction [12]. Briefly, four regions were manually segmented by different gray scale values of the microstructure and its morphological features. In OsO_4_-stained liver, the vessels had a lower gray scale value than that of parenchyma, indicating that veins and sinusoids can be distinguished from parenchyma [25]. Moreover, central vein and portal vein were distinguished by the presence of other branches of the portal tracts such as hepatic artery, bile duct, and lymphatic vessels [3]. In 3D volume-rendered images of liver tissues in normal and CCl_4_-treated mice, diameters, surface areas, and volumes of pericentral and periportal sinusoids were measured [8,12,26].

### Statistical analyses

All values are expressed as means ± S.D. *P* values were calculated from Student’s t tests, based on comparisons with the appropriate control samples tested at the same time.

## Results

### Identification of the sinusoid in normal liver

**Figure 1:**
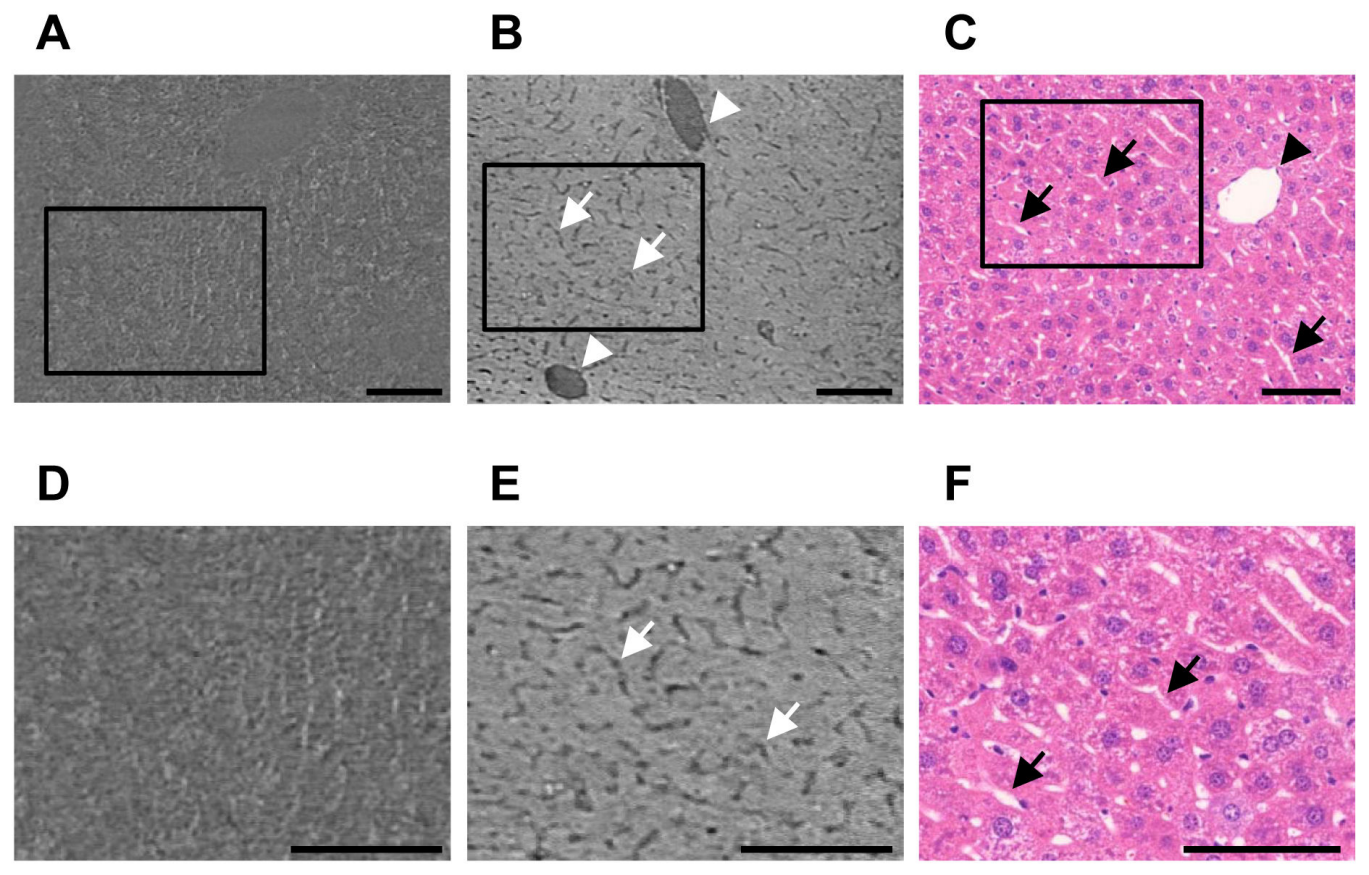
Tomographic slice and histological images of normal mouse liver. (A, D) Phase contrast image of mouse liver tissue obtained without OsO_4_ staining. The microvascular structure was ambiguous. (B, E) Absorption contrast image of mouse liver tissue with OsO_4_ staining. The sinusoids appeared as smaller pillars (arrows) and the veins seemed larger circles (arrowheads). (C, F) Histological image of normal mouse liver stained with hematoxylin and eosin (original magnification, ×20). (D–F) Magnification of the hepatic sinusoid region indicated by rectangles in Figure 1A, 1B, and 1C, respectively. Sinusoidal structure was similar between the histological images and absorption contrast X-ray images. Arrowheads indicate veins and arrows indicate sinusoids. Scale bar, 100 µm.

We performed synchrotron radiation micro-CT on normal liver tissue stained with or without an X-ray contrast agent, OsO_4_ (Figure 1). In the phase contrast images without OsO_4_ staining, the overall structure of the hepatic microvasculature was ambiguous, and we could not detect the hepatic sinusoids (Figure 1A and 1D). In absorption contrast images, we observed two different shapes of vessel-like structures on the OsO_4_-stained liver tissue (Figure 1B and 1E). One was circular with a diameter larger than 10 µm, and the other was rod-shaped with a diameter less than 10 µm. By comparing the representative tomographic slice image and the histological image stained with hematoxylin and eosin, we identified the vessel-like structure larger than 10 µm as a vein and one less than 10 µm as a sinusoid (Figure 1C and 1F). These data indicate that the absorption mode of synchrotron radiation micro-CT enables detection of the sinusoids. Additionally, based on the similar diameter and distribution of the sinusoids between the histological and X-ray images, the structures of the hepatic sinusoids were visualized by absorption contrast X-ray imaging.

### Identification of the sinusoids and veins in normal and CCl_4_-treated liver

**Figure 2:**
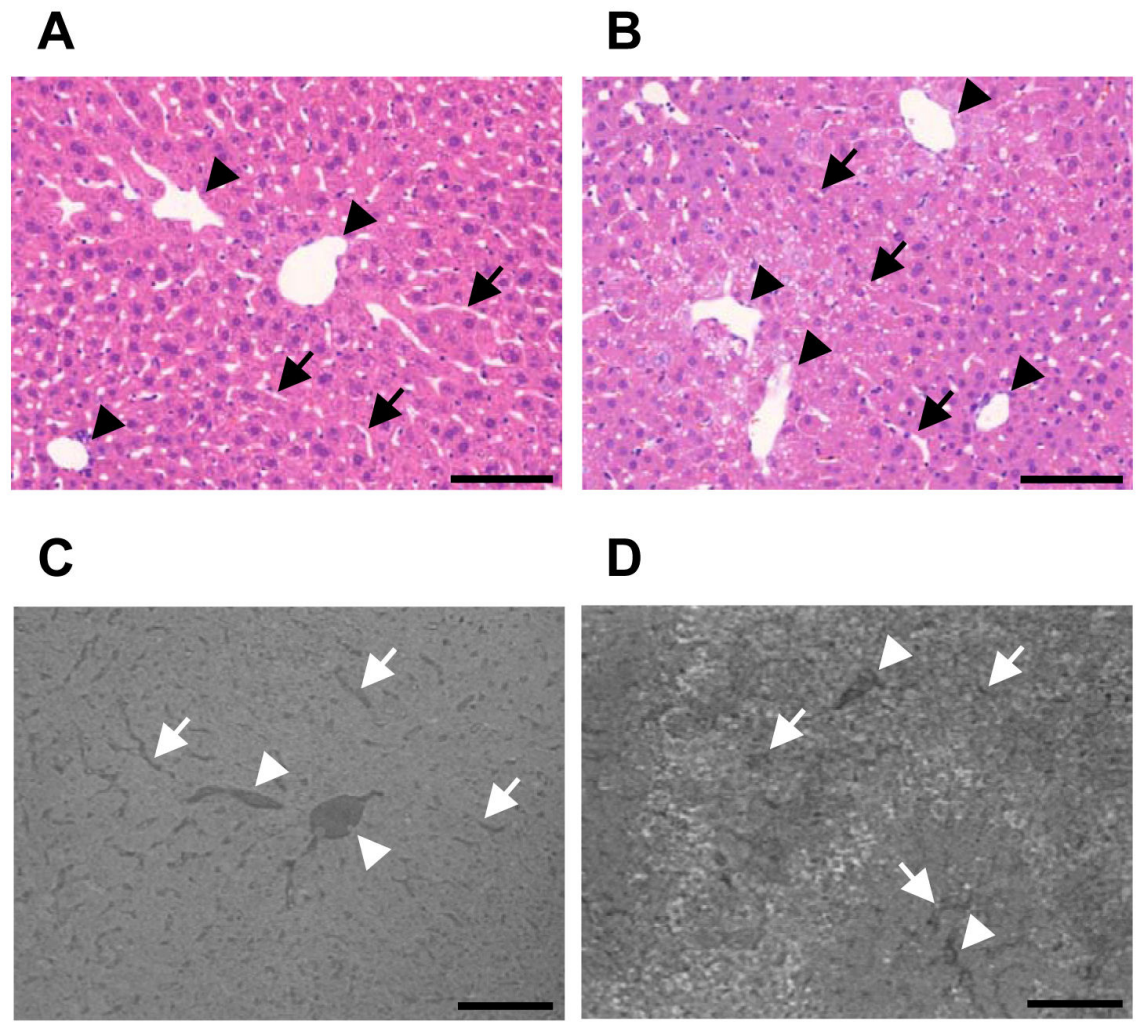
Tomographic slice and histological images of normal and acute CCl_4_-injured liver. Representative histological images of liver tissue section, stained with hematoxylin and eosin, (A) a normal mouse and (B) a mouse treated with CCl_4_ for 24 h. (original magnification, ×20). Representative coronal tomographic slice images of OsO_4_-stained (C) normal and (D) CCl_4_-treated mouse liver. Disrupted sinusoidal structures in necrotic areas were distinguished from non-necrotic regions from acute CCl_4_-injured mice. Arrowheads indicate veins and arrows indicate sinusoids. Scale bar, 100 µm.

We observed necrotic regions and disrupted sinusoidal structures in the histological images of normal and CCl_4_-treated liver (Figure 2A and 2B). We performed synchrotron radiation micro-CT on OsO_4_-stained liver tissue obtained from normal and CCl_4_-treated mice, and acquired the absorption contrast images (Figure 2C and 2D). Necrotic regions (hepatic sinusoids) could be distinguished from non-necrotic regions on the absorption contrast images from acute CCl_4_-injured mice. These findings suggest that structural alterations of the sinusoids in pathologically injured liver are detectable using the absorption mode of synchrotron radiation micro-CT. In particular, disease-specific pathological features such as necrosis and disrupted hepatic sinusoidal structures in necrotic areas were detected on absorption contrast images.

### Identification of pericentral sinusoid disruption in CCl_4_-treated liver

**Figure 3:**
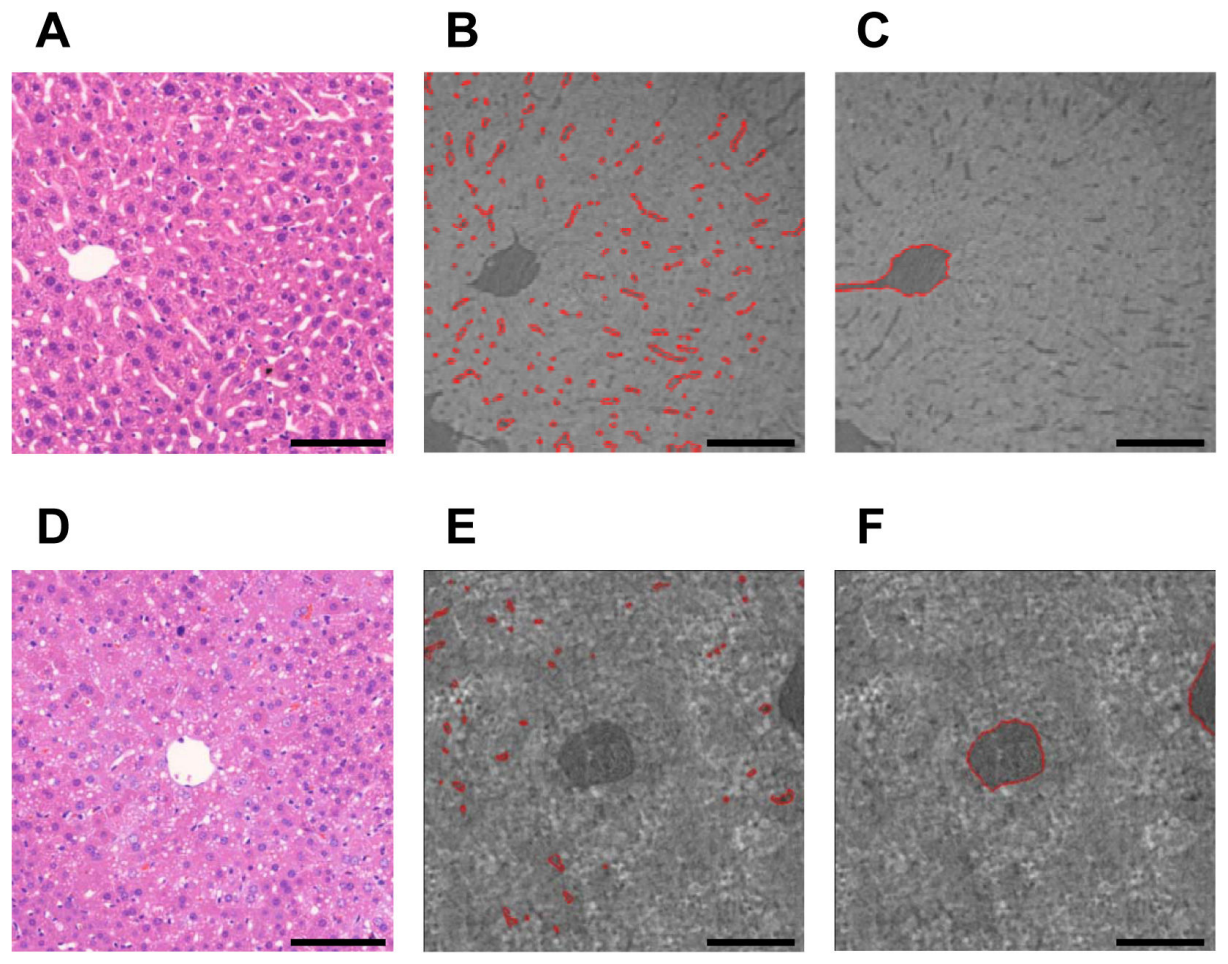
Tomographic slice and histological images of pericentral sinusoids and central vein. Pericentral sinusoids and central vein of livers from (A–C) normal and (D–F) CCl_4_-treated mice. Representative histological images of liver tissue, stained with hematoxylin and eosin, from (A) normal and (D) CCl_4_-treated mice (original magnification, ×20). Axial tomographic slice images of the pericentral area of OsO_4_-stained liver tissue segmented into the (B, E) pericentral sinusoids and (C, F) central vein, from normal and CCl_4_-treated mice. Solid red lines indicate segmented areas of the pericentral sinusoids (B, E) and of the central vein (C, F). Pericentral sinusoids were disrupted, but the central vein was unaffected by CCl_4_ damage in the CCl_4_-injured liver. Scale bar, 100 µm.

**Figure 4:**
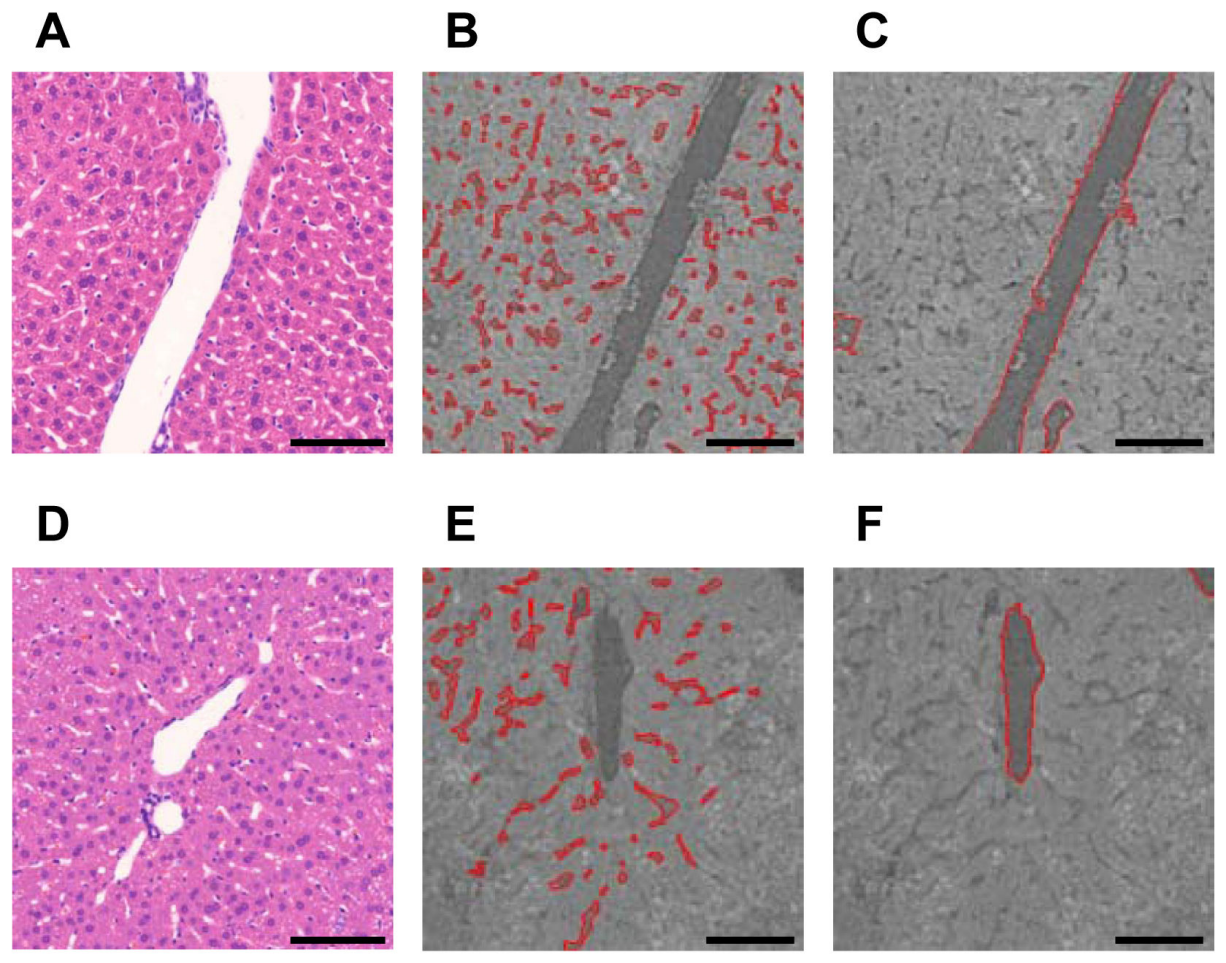
Tomographic slice and histological images of periportal sinusoids and portal vein. Periportal sinusoids and portal vein of livers (A–C) normal and (D–F) CCl_4_-treated mice. Representative histological images of liver tissue, stained with hematoxylin and eosin, from (A) normal and (D) CCl_4_-treated mice (original magnification, ×20). Coronal tomographic slice images of the pericentral area of OsO_4_-stained liver segmented into the (B, E) periportal sinusoids and (C, F) portal vein, from normal and CCl_4_-treated mice. Solid red lines indicate segmented areas of the periportal sinusoids (B, E) and of the portal vein (C, F). No significant difference in periportal sinusoids and the portal vein was observed between normal and CCl_4_-injured livers. Scale bar, 100 µm.

We next investigated the structural alterations of the hepatic sinusoids in the acute CCl_4_ model using the histological and absorption contrast X-ray images from the pericentral and periportal areas (Figures 3 and 4). Disrupted sinusoids in the necrotic region were detected in histological images of the pericentral area of CCl_4_-injured liver compared with normal liver (Figure 3A and 3D). When we segmented the pericentral sinusoids (Figure 3B and 3E) and the central vein (Figure 3C and 3F) in the absorption contrast images, we found that the sinusoidal structure was disrupted, but that the vein was protected from CCl_4_ damage in the CCl_4_-injured liver. No significant difference in the veins or sinusoids of the periportal area was observed between normal and CCl_4_-treated livers (Figure 4). Because we used a severe CCl_4_ injury model, the midzonal and some other sinusoids were disrupted, as shown in the absorption contrast X-ray images (Figure 4E and 4F). These data demonstrated that the particular sinusoidal alterations can be identified using the absorption mode of synchrotron radiation micro-CT.

### 3D structure of the sinusoids and veins in normal and CCl_4_-treated liver

**Figure 5:**
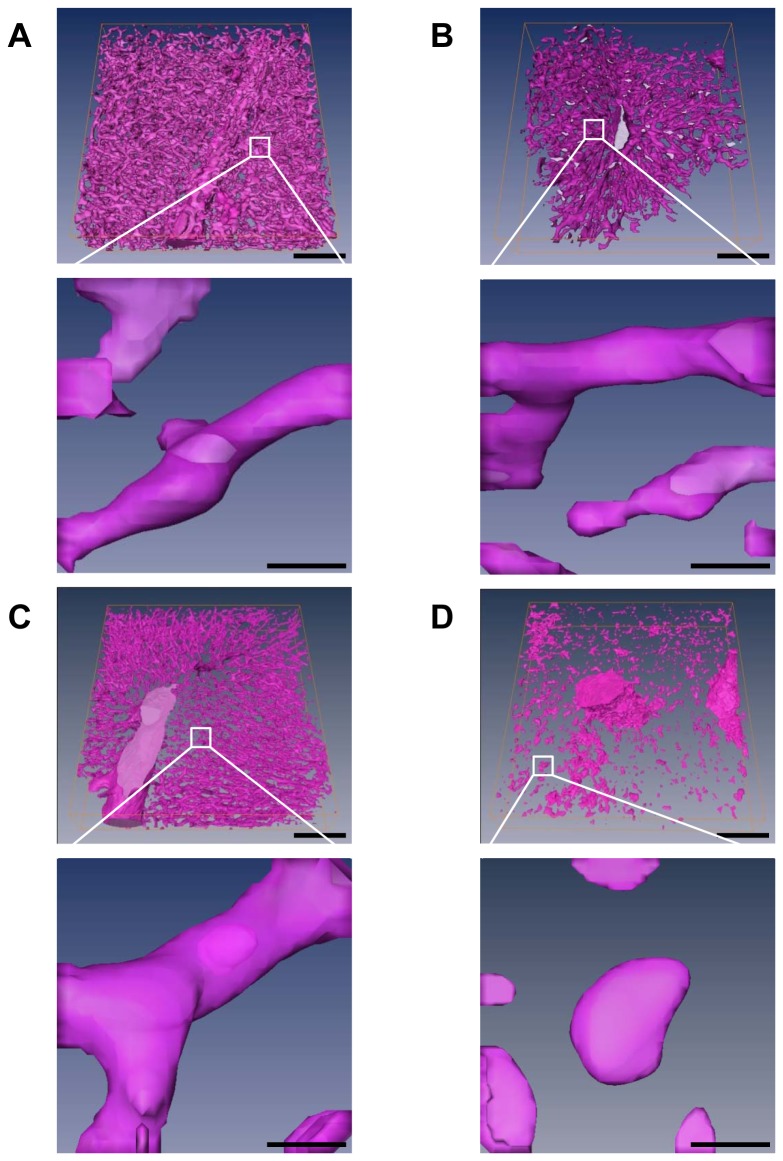
3D volume rendering images of hepatic sinusoids and veins. Volume rendered images of hepatic sinusoids and veins of (A, C) normal and (B, D) CCl_4_-treated mice. Three-dimensional images shown according to metabolic zonation of the liver lobule: (A, B) periportal area and (C, D) pericentral area. The portion of the white box was enlarged to show the magnified view of 3D volume-rendered images of periportal and pericentral sinusoids in normal and CCl_4_-treated mice. The size of the 3D image is 435 × 435 × 145 µm^3^ before magnification and 36 × 36 × 36 µm^3^ after magnification. Disrupted 3D structures of pericentral sinusoids were distinguished from undisrupted 3D periportal sinusoids in CCl_4_-injured liver. Scale bars, 100 µm. Enlarged images scale bars, 10 µm.

We next investigated the 3D structure of the hepatic sinusoids and veins in normal and CCl_4_-treated mice (Figure 5). No significant difference in the sinusoidal structure in the periportal area was observed between normal and acutely injured mice (Figure 5A and 5B). In contrast, the 3D image of the pericentral sinusoids in CCl_4_-treated mice showed sparser and more disconnected vascular network compared with that in the normal mice (Figure 5C and 5D). Thus, disrupted structures of pericentral sinusoids can be distinguished from undisrupted periportal sinusoids in 3D images of the hepatic sinusoidal network of acute CCl_4_-injured liver, as observed by synchrotron radiation micro-CT.

### Quantitative analysis of hepatic sinusoids in normal and CCl_4_-treated liver

**Figure 6:**
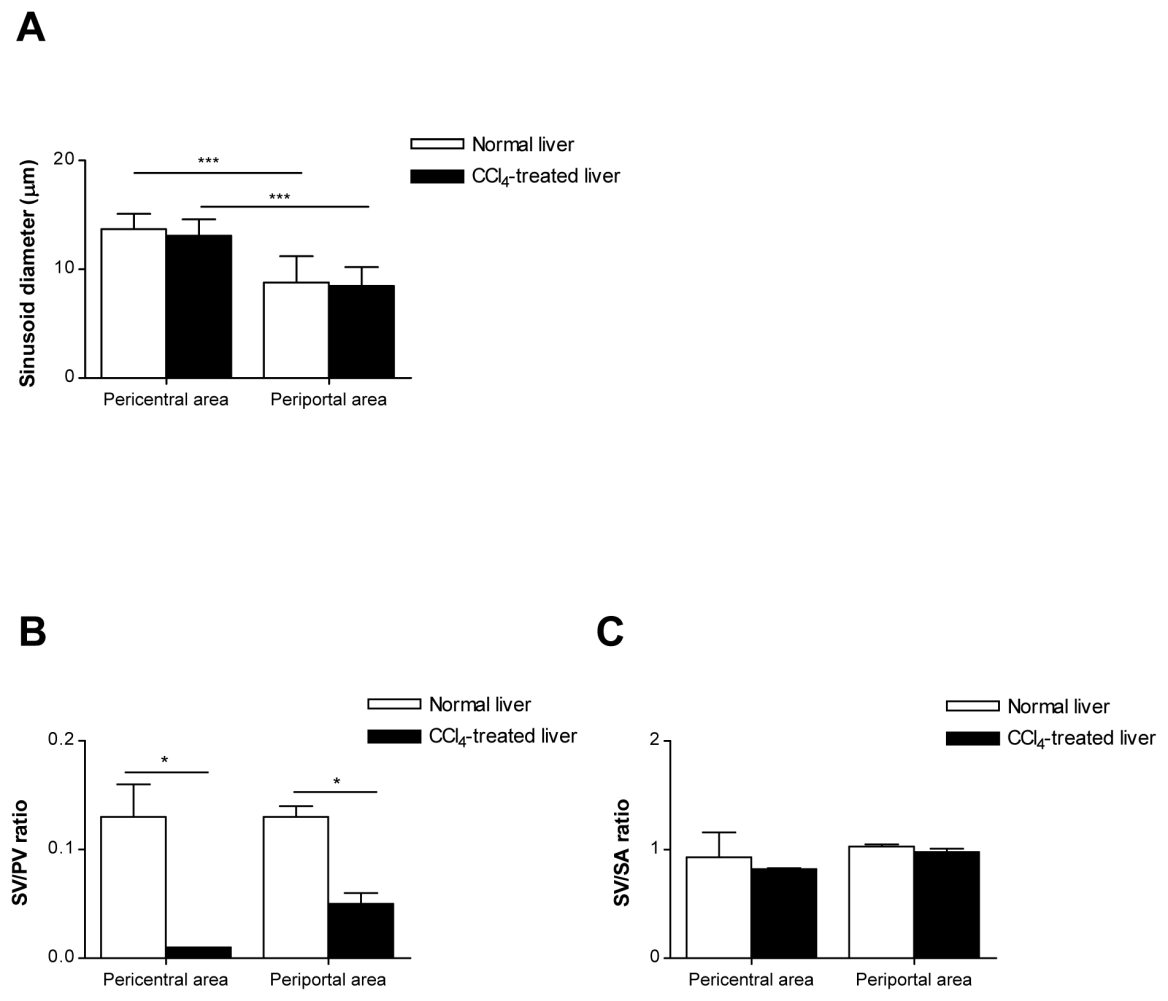
Quantitative analysis of hepatic sinusoids in normal and CCl_4_-treated liver. (A) Sinusoidal diameters in pericentral and periportal regions were measured in normal and CCl_4_-treated mice. (B) The sinusoidal volume (SV)/parenchymal volume (PV) ratio and (C) sinusoidal volume (SV)/sinusoidal surface area (SA) ratio were measured in normal and CCl_4_-treated mice. Data are represented as mean ± SD. *P< 0.05; ***P< 0.001.

In 3D volume-rendered images (Figure 5), diameters of pericentral and periportal sinusoids were measured in normal and CCl_4_-treated liver (Figure 6A). In normal liver, sinusoidal diameters of pericentral and periportal area were 13.7 ± 1.4 µm and 8.8 ± 2.4 µm, respectively. The diameter of pericentral sinusoid was larger than that of periportal sinusoids. After acute CCl_4_ treatment, there was no significant difference in the diameter of periportal and pericentral sinusoids compared with control (Figure 6A).

We also performed a quantitative analysis on volume-rendered 3D image of normal and CCl_4_-treated liver using morphological parameter including surface area and volume. The results of measured surface area and volume of the sinusoid and parenchyma in normal and CCl_4_-treated liver are given in Table 1. The total volume of the parenchyma was almost the same in normal and CCl_4_-treated liver (both about 20.2 × 10^6^ µm^3^); whereas the volume of the sinusoid differed; 2.64 ± 0.54 × 10^6^ µm^3^ (pericentral area) and 2.60 ± 0.27 × 10^6^ µm^3^ (periportal area) in normal liver and 0.28 ± 0.05 × 10^6^ µm^3^ (pericentral area) and 0.96 ± 0.25 × 10^6^ µm^3^ (periportal area) in CCl_4_-treated liver. The sinusoidal volume/parenchymal volume ratio in periportal and pericentral area was significantly lower in CCl_4_-treated mice compared with control, indicating the reduction in the sinusoidal volume fraction by acute CCl_4_ treatment (Figure 6B). However, no significant difference was observed in the sinusoidal volume/sinusoidal surface area ratio between normal and CCl_4_-treated mice (Figure 6C). The low value of sinusoidal volume/sinusoidal surface area indicates a decrease in the number of sinusoids as the diameter of hepatic sinusoids appeared to be similar.

**Table 1:** Quantitative assessment on volume-rendered 3D images from normal and CCl_4_-treated livers.

	Surface Area (×10^6^ µm^2^)		Sinusoidal Volume (×10^6^ µm^3^)		Parenchymal Volume (×10^6^ µm^3^)	
	Normal	CCl_4_	Normal	CCl_4_	Normal	CCl_4_
Pericentral Area	2.87 ± 0.32	0.34 ± 0.07**	2.64 ± 0.54	0.28 ± 0.05*	20.2 ± 0.07	20.2 ± 0.00
Periportal Area	2.52 ± 0.21	0.97 ± 0.22*	2.60 ± 0.27	0.96 ± 0.25*	20.2 ± 0.00	20.2 ± 0.00

Values are mean ± SD. * *P*< 0.05; ** *P*< 0.01 compared to normal.

## Discussion

We demonstrated that the absorption mode of synchrotron radiation micro-CT can produce 3D images of hepatic sinusoids and their alterations in normal and injured mouse livers. Furthermore, pathological features of acute liver injury, such as the disruption of pericentral sinusoids in necrotic regions, were detectable in the 3D images from synchrotron radiation micro-CT.

Synchrotron radiation X-rays generally use absorption or phase contrast effects when visualizing the angioarchitecture of tissue [18–20,27]. Synchrotron radiation X-rays with high coherence can induce a phase contrast effect through edge enhancement [20,27]. In absorption mode, the angioarchitecture of the tissue can be visualized by microinjecting an X-ray contrast agent into the vessels or staining the surrounding tissues. As conventional X-ray contrast agents, including radiopaque polymers, fill only the lumen side of the blood vessel, the vascular architecture and the interrelationships among vessels are difficult to visualize [15,25,28,29]. Recently, Ritman et al. suggested the use of OsO_4_ as an X-ray contrast agent for micro-CT because it enhanced visualization of the coronary artery wall structure and nephron architecture, and their interrelationships [25,29]. We visualized the 3D hepatic sinusoidal structures using the absorption mode of synchrotron radiation micro-CT with OsO_4_ as an X-ray contrast agent. The presence of OsO_4_ resulted in lower absorption by vessels than by parenchyma in the absorption contrast images of mouse livers.

The liver, which consists of many structural and functional units called lobules, is the main metabolic organ in the body [1]. Detoxification is conducted in each lobule of the liver, and thus understanding microvascular alterations of the lobule in three-dimensions during liver injury and regeneration is significant. Several imaging techniques have been applied to visualize the angioarchitecture of the lobule at 3D space in order to elucidate the microvasculature of the liver lobule under physiological conditions [11,13,14]. Although light or confocal microscopy has sufficient spatial resolution to visualize the sinusoids, the penetration into thick specimens is limited. Thus, light and confocal microscopic studies on the liver require thin tissue sections, so the depth of the observable tissue is restricted. The 3D angioarchitecture of the lobule has been investigated at the level of the sinusoids using confocal laser scanning microscopy, but the depth of the specimen is limited to about 150 µm thick liver tissue slices [11]. In contrast, we observed the 3D angioarchitecture of the liver lobule at the level of the sinusoids with liver tissue sections of 2-3 mm thickness using synchrotron radiation micro-CT. Moreover, synchrotron radiation X-rays with a spatial resolution of 1 µm is sufficient to detect the sinusoids with diameters of 7-15 µm in the mouse liver. In our study, we observed that hepatic sinusoids in the normal liver were directly branched off from the portal veins [3,4]. Moreover, hepatic sinusoids were running between the liver cells and interconnecting sinusoids were also present between the sinusoids [4]. In addition, consistent with previous reports [3,4], sinusoidal diameters of pericentral and periportal area were 13.7 ± 1.4 µm and 8.8 ± 2.4 µm, respectively, and pericentral sinusoids were larger than periportal sinusoids.

In both the two-dimensional and 3D images of synchrotron radiation micro-CT, we distinguished disrupted pericentral sinusoids from undisrupted periportal sinusoids in the acute CCl_4_-injured liver. Injury to the hepatic microvasculature induced by CCl_4_ occurs via the cytochrome P450s [30]. These enzymes reside in the parenchyma and generate a concentration gradient from the central to the portal vein. Thus, CCl_4_ treatment causes necrosis of pericentral sinusoids. OsO_4_ strongly binds to the lipids, and CCl_4_ induces lipid alterations in necrotic regions [31]. Therefore, when OsO_4_ is used as an X-ray contrast agent, the absorption contrast of necrotic regions (pericentral sinusoids) should be higher than that of non-necrotic regions (periportal sinusoids) in acute CCl_4_-injured liver. Recent studies on structural alteration of hepatic sinusoid in acute CCl_4_ model have been conducted by light microscopy, confocal laser microscopy, and intravital fluorescence microscopy [9–11,26]. After acute treatment with CCl_4_, sinusoidal diameter was significantly smaller than that in controls and sinusoidal area was significantly reduced in CCl_4_-treated mice [9,26]. Moreover, pericentral sinusoids are scattered in the necrotic region [10,11]. Our results of 3D volume rendering image were identical to the previous studies in that the volume and the vessel connectivity of the pericentral sinusoid were significantly reduced and disconnected, respectively [9,10]. Because our model was acute CCl_4_ injury model, we were not able to observe the fibrotic and cirrhotic region of the liver, observed in chronic CCl_4_ injury model [8,32]. These fibrotic and cirrhotic regions can be observed using our system of synchrotron radiation micro-CT combined with OsO_4_ staining. Therefore, in the future, we are planning to visualize three-dimensionally, the extensive fibrotic and cirrhotic regions of the chronic liver models in both mouse and human liver.

For decades, structural alterations of hepatic sinusoidal endothelial cells having sieve plate-like pores have been studied by scanning and transmission electron microscopy [3,4,6]. The limitation of our study in terms of anatomical imaging was that the spatial resolution of synchrotron radiation micro-CT is not sufficient to investigate the unique morphology of the hepatic sinusoidal endothelial cells [6]. Synchrotron radiation micro-CT, on the other hand, is a feasible imaging system to investigate the diameter and distribution of hepatic sinusoids rather than the structural alterations of hepatic sinusoidal endothelial cells. Further improvement of this imaging system with a subnano spatial resolution will make possible the 3D visualization of characteristic morphology of sinusoidal endothelial cells in normal and pathological conditions.

In our study, to visualize the hepatic sinusoid, liver tissues had to be postfixed and stained in OsO_4_ to give an absorption contrast and embedded in resin to protect the tissue from high flux X-rays. This fixation and staining made simultaneous functional imaging of the liver difficult in our system [23,29]. In addition, in the processes of fixation, postfixation with OsO_4_, and dehydration, the shrinkage and the collapse of morphological structure in the specimen can be induced [33]. However, we could not detect any artifacts in the overall structure of liver tissue when our results were compared with histological images. The combination of histological and confocal imaging of the liver tissues with synchrotron radiation X-ray microscopy can be applied to further examine the possible artifacts [11,20].

In conclusion, we nondestructively identified the hepatic sinusoids and their structural alterations in acutely injured mouse liver using the absorption mode of synchrotron radiation micro-CT for the first time. The 3D structure and their alterations of the sinusoids could be identified in diverse hepatic diseases including fibrosis, cirrhosis and liver cancer in human.

## References

[1] Taub R (2004) Liver regeneration: from myth to mechanism. Nat Rev Mol Cell Biol 5: 836-847. doi:10.1038/nrm1489. PubMed: 15459664. 15459664 10.1038/nrm1489

[2] Rappaport AM, Black RG, Lucas CC, Ridout JH, Best CH (1966) Normal and pathologic microcirculation of the living mammalian liver. Rev Int Hepatol 16: 813-828. PubMed: 5919731. 5919731

[3] Vollmar B, Menger MD (2009) The hepatic microcirculation: mechanistic contributions and therapeutic targets in liver injury and repair. Physiol Rev 89: 1269-1339. doi:10.1152/physrev.00027.2008. PubMed: 19789382. 19789382 10.1152/physrev.00027.2008

[4] Oda M, Yokomori H, Han JY (2003) Regulatory mechanisms of hepatic microcirculation. Clin Hemorheol Microcirc 29: 167-182. PubMed: 14724338. 14724338

[5] Fraser R, Dobbs BR, Rogers GW (1995) Lipoproteins and the liver sieve: the role of the fenestrated sinusoidal endothelium in lipoprotein metabolism, atherosclerosis, and cirrhosis. Hepatology 21: 863-874. doi:10.1016/0270-9139(95)90542-1. PubMed: 7875685. 7875685

[6] Braet F, Wisse E (2002) Structural and functional aspects of liver sinusoidal endothelial cell fenestrae: a review. Comp Hepatol 1: 1. doi:10.1186/1476-5926-1-1. PubMed: 12437787. 12437787 10.1186/1476-5926-1-1 PMC131011

[7] Sarin H (2010) Physiologic upper limits of pore size of different blood capillary types and another perspective on the dual pore theory of microvascular permeability. J Angiogenes Res 2: 14. doi:10.1186/2040-2384-2-14. PubMed: 20701757. 20701757 10.1186/2040-2384-2-14 PMC2928191

[8] Onori P, Morini S, Franchitto A, Sferra R, Alvaro D et al. (2000) Hepatic microvascular features in experimental cirrhosis: a structural and morphometrical study in CCl4-treated rats. J Hepatol 33: 555-563. doi:10.1034/j.1600-0641.2000.033004555.x. PubMed: 11059860. 11059860 10.1034/j.1600-0641.2000.033004555.x

[9] Cao AH, Vo LT, King RG (2005) Honokiol protects against carbon tetrachloride induced liver damage in the rat. Phytother Res 19: 932-937. doi:10.1002/ptr.1757. PubMed: 16317648. 16317648 10.1002/ptr.1757

[10] Yoneyama H, Kai Y, Koyama J, Suzuki K, Kawachi H et al. (2007) Neutralization of CXCL10 accelerates liver regeneration in carbon tetrachloride-induced acute liver injury. Med Mol Morphol 40: 191-197. doi:10.1007/s00795-007-0371-x. PubMed: 18085377. 18085377 10.1007/s00795-007-0371-x

[11] Hoehme S, Brulport M, Bauer A, Bedawy E, Schormann W et al. (2010) Prediction and validation of cell alignment along microvessels as order principle to restore tissue architecture in liver regeneration. Proc Natl Acad Sci U S A 107: 10371-10376. doi:10.1073/pnas.0909374107. PubMed: 20484673. 20484673 10.1073/pnas.0909374107 PMC2890786

[12] Duan J, Hu C, Chen H (2013) High-resolution micro-CT for morphologic and quantitative assessment of the sinusoid in human cavernous hemangioma of the liver. PLOS ONE 8: e53507. doi:10.1371/journal.pone.0053507. PubMed: 23308240. 23308240 10.1371/journal.pone.0053507 PMC3538536

[13] Teutsch HF, Schuerfeld D, Groezinger E (1999) Three-dimensional reconstruction of parenchymal units in the liver of the rat. Hepatology 29: 494-505. doi:10.1002/hep.510290243. PubMed: 9918927. 9918927 10.1002/hep.510290243

[14] Teutsch HF (2005) The modular microarchitecture of human liver. Hepatology 42: 317-325. doi:10.1002/hep.20764. PubMed: 15965926. 15965926 10.1002/hep.20764

[15] Masyuk TV, Ritman EL, LaRusso NF (2003) Hepatic artery and portal vein remodeling in rat liver: vascular response to selective cholangiocyte proliferation. Am J Pathol 162: 1175-1182. doi:10.1016/S0002-9440(10)63913-2. PubMed: 12651609. 12651609 10.1016/S0002-9440(10)63913-2 PMC1851241

[16] Masyuk TV, Huang BQ, Masyuk AI, Ritman EL, Torres VE et al. (2004) Biliary dysgenesis in the PCK rat, an orthologous model of autosomal recessive polycystic kidney disease. Am J Pathol 165: 1719-1730. doi:10.1016/S0002-9440(10)63427-X. PubMed: 15509540. 15509540 10.1016/S0002-9440(10)63427-X PMC1618661

[17] Op Den Buijs J, Bajzer Z, Ritman EL (2006) Branching morphology of the rat hepatic portal vein tree: a micro-CT study. Ann Biomed Eng 34: 1420-1428. doi:10.1007/s10439-006-9150-4. PubMed: 16838126. 16838126 10.1007/s10439-006-9150-4

[18] Meuli R, Hwu Y, Je JH, Margaritondo G (2004) Synchrotron radiation in radiology: radiology techniques based on synchrotron sources. Eur Radiol 14: 1550-1560. PubMed: 15316744. 15316744 10.1007/s00330-004-2361-x

[19] Plouraboue F, Cloetens P, Fonta C, Steyer A, Lauwers F et al. (2004) X-ray high-resolution vascular network imaging. J Microsc 215: 139-148. doi:10.1111/j.0022-2720.2004.01362.x. PubMed: 15315500. 15315500 10.1111/j.0022-2720.2004.01362.x

[20] Heinzer S, Krucker T, Stampanoni M, Abela R, Meyer EP et al. (2006) Hierarchical microimaging for multiscale analysis of large vascular networks. NeuroImage 32: 626-636. doi:10.1016/j.neuroimage.2006.03.043. PubMed: 16697665. 16697665 10.1016/j.neuroimage.2006.03.043

[21] Simeonova PP, Gallucci RM, Hulderman T, Wilson R, Kommineni C et al. (2001) The role of tumor necrosis factor-alpha in liver toxicity, inflammation, and fibrosis induced by carbon tetrachloride. Toxicol Appl Pharmacol 177: 112-120. doi:10.1006/taap.2001.9304. PubMed: 11740910. 11740910 10.1006/taap.2001.9304

[22] Donthamsetty S, Bhave VS, Mitra MS, Latendresse JR, Mehendale HM (2007) Nonalcoholic fatty liver sensitizes rats to carbon tetrachloride hepatotoxicity. Hepatology 45: 391-403. doi:10.1002/hep.21530. PubMed: 17256749. 17256749 10.1002/hep.21530

[23] Hwu Y, Tsai WL, Chang HM, Yeh HI, Hsu PC et al. (2004) Imaging cells and tissues with refractive index radiology. Biophys J 87: 4180-4187. doi:10.1529/biophysj.103.034991. PubMed: 15465870. 15465870 10.1529/biophysj.103.034991 PMC1304927

[24] Kim JW, Seo HS, Hwu Y, Je JH, Kim A et al. (2007) In vivo real-time vessel imaging and ex vivo 3D reconstruction of atherosclerotic plaque in apolipoprotein E-knockout mice using synchrotron radiation microscopy. Int J Cardiol 114: 166-171. doi:10.1016/j.ijcard.2005.12.010. PubMed: 16831476. 16831476 10.1016/j.ijcard.2005.12.010

[25] Bentley MD, Jorgensen SM, Lerman LO, Ritman EL, Romero JC (2007) Visualization of three-dimensional nephron structure with microcomputed tomography. Anat Rec (Hoboken) 290: 277-283. doi:10.1002/ar.20422. PubMed: 17525936. 17525936 10.1002/ar.20422

[26] Vanheule E, Geerts AM, Reynaert H, Van Vlierberghe H, Geerts A et al. (2008) Influence of somatostatin and octreotide on liver microcirculation in an experimental mouse model of cirrhosis studied by intravital fluorescence microscopy. Liver Int 28: 107-116. PubMed: 18173562. 18173562 10.1111/j.1478-3231.2007.01629.x

[27] Momose A, Takeda T, Itai Y (2000) Blood vessels: depiction at phase-contrast X-ray imaging without contrast agents in the mouse and rat-feasibility study. Radiology 217: 593-596. PubMed: 11058666. 11058666 10.1148/radiology.217.2.r00oc14593

[28] Bentley MD, Ortiz MC, Ritman EL, Romero JC (2002) The use of microcomputed tomography to study microvasculature in small rodents. Am J Physiol Regul Integr Comp Physiol 282: R1267-R1279. PubMed: 11959666. 11959666 10.1152/ajpregu.00560.2001

[29] Zhu XY, Bentley MD, Chade AR, Ritman EL, Lerman A et al. (2007) Early changes in coronary artery wall structure detected by microcomputed tomography in experimental hypercholesterolemia. Am J Physiol Heart Circ Physiol 293: H1997-H2003. doi:10.1152/ajpheart.00362.2007. PubMed: 17573460. 17573460 10.1152/ajpheart.00362.2007

[30] Oinonen T, Lindros KO (1998) Zonation of hepatic cytochrome P-450 expression and regulation. Biochem J 329(1): 17-35. PubMed: 9405271. 9405271 10.1042/bj3290017 PMC1219009

[31] Martínez-Calva I, Campos-Apáez A, Rosales-Vega E, Mourelle M (1984) Vitamin E improves membrane lipid alterations induced by CCl4 intoxication. J Appl Toxicol 4: 270-272. doi:10.1002/jat.2550040513. PubMed: 6512166. 6512166 10.1002/jat.2550040513

[32] Iredale JP (2003) Cirrhosis: new research provides a basis for rational and targeted treatments. Bmj 327: 143-147. doi:10.1136/bmj.327.7422.s143. PubMed: 12869458. 12869458 10.1136/bmj.327.7407.143 PMC1126509

[33] Weston AE, Armer HE, Collinson LM (2009) Towards native-state imaging in biological context in the electron microscope. Chem Biol 3: 101-112. PubMed: 19916039. 10.1007/s12154-009-0033-7 PMC2906717 19916039

